# Progesterone Inhibits the Establishment of Activation-Associated Chromatin During T_H_1 Differentiation

**DOI:** 10.3389/fimmu.2022.835625

**Published:** 2022-02-02

**Authors:** Olof Rundquist, Colm E. Nestor, Maria C. Jenmalm, Sandra Hellberg, Mika Gustafsson

**Affiliations:** ^1^ Bioinformatics, Department of Physics, Chemistry and Biology, Linköping University, Linköping, Sweden; ^2^ Crown Princess Victoria Children’s Hospital, and Department of Biomedical and Clinical Sciences, Linköping University, Linköping, Sweden; ^3^ Division of Inflammation and Infection, Department of Biomedical and Clinical Sciences, Linköping University, Linköping, Sweden

**Keywords:** T_H_1, progesterone, T-cell activation, ATAC-seq, transcriptomics, T_H_1- mediated diseases

## Abstract

T_H_1-mediated diseases such as multiple sclerosis (MS) and rheumatoid arthritis (RA) improve during pregnancy, coinciding with increasing levels of the pregnancy hormone progesterone (P4), highlighting P4 as a potential mediator of this immunomodulation. Here, we performed detailed characterization of how P4 affects the chromatin and transcriptomic landscape during early human T_H_1 differentiation, utilizing both ATAC-seq and RNA-seq. Time series analysis of the earlier events (0.5-24 hrs) during T_H_1 differentiation revealed that P4 counteracted many of the changes induced during normal differentiation, mainly by downregulating key regulatory genes and their upstream transcription factors (TFs) involved in the initial T-cell activation. Members of the AP-1 complex such as FOSL1, FOSL2, JUN and JUNB were particularly affected, in both in promoters and in distal regulatory elements. Moreover, the changes induced by P4 were significantly enriched for disease-associated changes related to both MS and RA, revealing several shared upstream TFs, where again JUN was highlighted to be of central importance. Our findings support an immune regulatory role for P4 during pregnancy by impeding T-cell activation, a crucial checkpoint during pregnancy and in T-cell mediated diseases, and a central event prior to T-cell lineage commitment. Indeed, P4 is emerging as a likely candidate involved in disease modulation during pregnancy and further studies evaluating P4 as a potential treatment option are needed.

## Introduction

T helper (T_H_) 1 cells, through the action of the signature cytokine IFN-γ, are crucial for defence against intracellular pathogens (1). However, inappropriate activation and aberrant regulation of T_H_1 responses can be detrimental and have been associated with autoimmune diseases like multiple sclerosis (MS) and rheumatoid arthritis (RA) (2, 3). Interestingly, T_H_1-mediated diseases are known to improve during pregnancy (4, 5) and the rising levels of the pregnancy hormone progesterone (P4) has been suggested to be involved in the disease modulation. Indeed, exposure of T cells to P4 has previously been shown to modulate T_H_1 responses, mainly through dampening of T_H_1-associated cytokine secretion (6–9). Furthermore, P4 has been shown to limit T cell activation and proliferation (10, 11), possibly impeding the action of autoreactive T_H_1 cells. A deeper understanding of how P4 influences T_H_1 activation and differentiation could provide further insights into its role in disease modulation during pregnancy and potential as a novel therapy in T_H_1-related autoimmune diseases.

T_H_1 differentiation, upon antigen recognition through the T cell receptor, is initiated by the actions of IL-12 and IFN-y through STAT1 and STAT4 signalling, which in turn leads to T_H_1 commitment by promoting the expression of TBX21, the master regulator of T_H_1 cells (12–14). T_H_1 differentiation involves an orchestrated, step-wise regulation of interactions between transcription factors (TFs) and genes. Although TFs are clearly vital players for the transcription of lineage-specific genes that drive the differentiation of one T_H_ subset over the other, gene expression is more complex than the binding of a single TF to a single target DNA sequence. As the genome is packaged into chromatin, the relative accessibility of the chromatin also constitutes an important aspect of gene regulation (15, 16). Open chromatin allows for access of *cis*-regulatory elements like promotors and enhancers by the transcription machinery while closed chromatin regions prevent access and thereby transcription. Changes in the chromatin landscape, for example due to naturally occurring mutations, could prevent or promote chromatin interactions at TF binding sites, which in turn could promote pathogenic gene expression profiles, predisposing for autoimmune diseases. A deeper knowledge of the gene regulatory landscape underlying gene expression and cellular profiles is important for understanding disease pathogenesis. For this purpose, the assay for transposase-accessible chromatin using sequencing (ATAC-seq) has emerged as a powerful tool for investigating genome-wide chromatin accessibility profiling (17).

Here we performed ATAC-seq and RNA-seq on time series data during early T_H_1 differentiation in the absence or presence of P4 to define the chromatin and related transcriptomic changes underlying T_H_1 differentiation and how P4 affects the gene regulatory landscape of T_H_1 cells. A better understanding of the underlying processes involved in shaping T_H_1 cells are a pre-requisite for understanding diseases driven by these cells. Further, identifying factors, such as P4, that could potentially modulate this process could enable new treatment strategies of T_H_1-mediated diseases. This study expands on the previously known immune regulatory effects of P4, highlighting a potential role for P4 in T_H_1-mediated diseases that are known to improve during pregnancy when P4 levels are high.

## Materials and Methods

### Ethics Statement

Buffy coats were purchased from the blood bank facility at Linköping University Hospital, where the blood was collected from healthy donors. The blood donors had given written consent for research use (besides medical use) of the donated blood in accordance with the Declaration of Helsinki. Since blood donation is classified as a negligible risk to the donors and since only deidentified samples were delivered, the use of the samples does not require a specific ethical approval according to paragraph 4 of the Swedish law (2003∶460) on Ethical Conduct in Human Research.

### Isolation of Naïve CD4+ T Cells

Venous blood was collected in buffy coats purchased from the blood bank at Linköping University Hospital from a total of 3 healthy female donors. Donors gave written informed consent for research use of the blood. Peripheral blood mononuclear cells (PBMCs) were then isolated by density gradient centrifugation with Lymphoprep™ (Axis Shields Diagnostics, Dundee, Scotland). Following washing in phosphate buffer saline (PBS), the PBMCs were resuspended in MACS buffer (PBS supplemented with fetal bovine serum (FBS, heat-inactivated; Gibco^®^, ThermoFisher Scientific, Waltham, Massachusetts, USA) and 2mM EDTA) and CD45RA^+^ CD4^+^ T cells were subsequently isolated with negative immunomagnetic selection using an LS column and a MidiMACS™ separator according to the instructions provided by the manufacturer for the “Naive CD4+ T cell Isolation Kit II, human (Miltenyi Biotec, Bergisch Gladbach, Germany).

### Pre-Incubation With Progesterone

The isolated naïve CD4^+^ were cultured in 6-well TPP^®^ tissue culture plates (Sigma-Aldrich, Saint Louis, MO, USA; 1 million cells/ml) and incubated overnight, 37°C and 5% CO_2_ with either mock treatment (cell culture media alone) or 50 µM P4 (water-soluble; Sigma Aldrich). After 24 hours, the cells were removed from the plates, centrifuged, and resuspended in fresh growth media.

### T_H_1 Differentiation in the Absence or Presence of Progesterone

**Figure 1:**
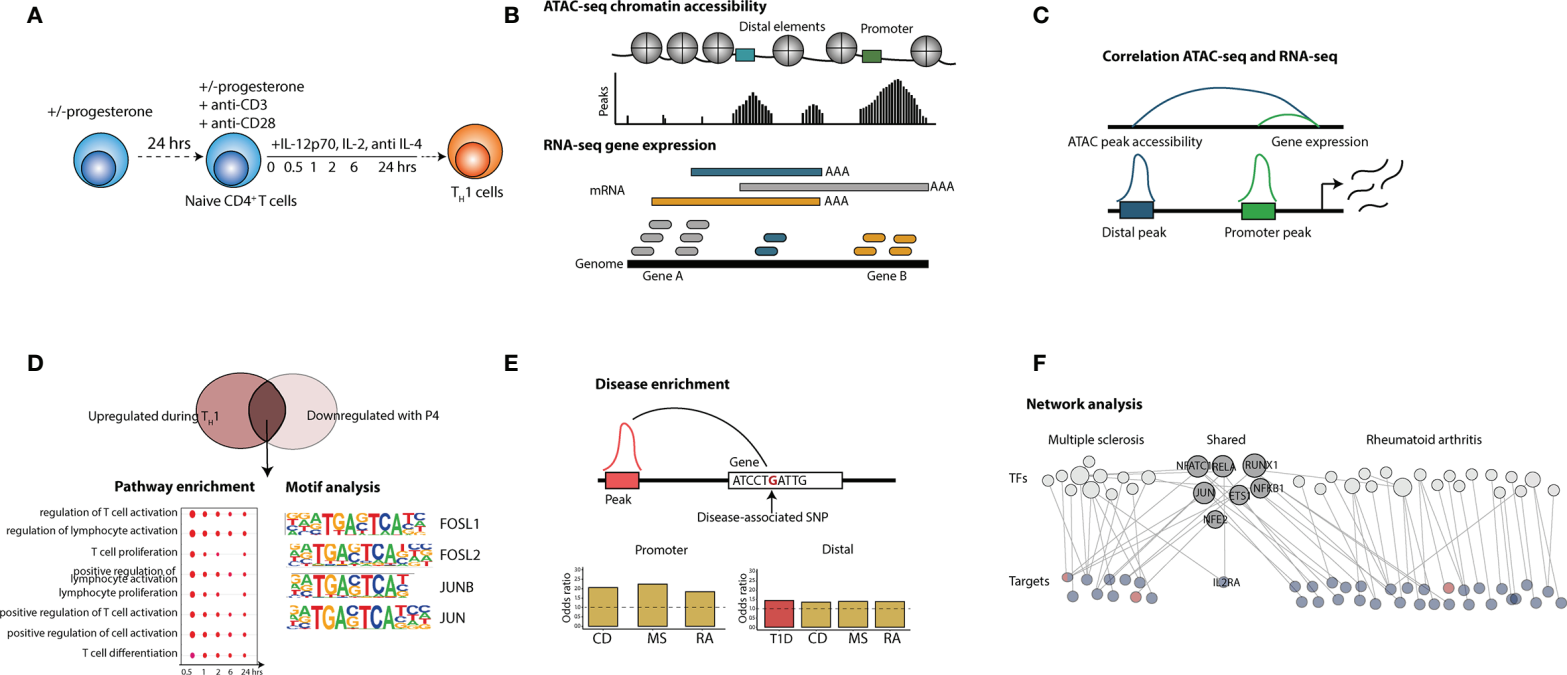
Overview of the study. The figure shows the principal steps of the different analysis performed in the study. **(A)** Isolated naïve CD4^+^ T cells were activated with anti-CD3/28 beads in the presence of T_H_1 polarizing factors (IL-12p70, IL-2 and anti-IL-4) for 0.5, 1,2, 6 and 24 hrs with or without P4 (n = 3). Naïve T cells cultured together with 50 µM P4 had also been pre-incubated with P4 for 24 hrs. **(B)** ATAC-sequencing to evaluate chromatin accessibility and RNA-seq for gene expression analysis were used as read out. **(C)** ATAC-seq and RNA-data was integrated through correlation where peak accessibility for promoter and distal peaks was correlated to changes in gene expression. **(D)** Peaks and genes upregulated during T_H_1 differentiation were overlapped with peaks and genes downregulated by P4 and the intersecting changes were analyzed for pathway enrichment and transcription factor (TF) motif analysis. **(E)** The intersecting peaks were also associated to genes containing disease-SNPs for a wide array of different types of diseases and analyzed for disease enrichment. **(F)** Disease-associated changes connecting to changes induced by P4 were used to construct a network of disease-associated transcription factors and target genes for both MS and RA. CD, Crohn’s disease; MS, multiple sclerosis; SNP, single nucleotide polymorphism; TF, transcription factor; RA, rheumatoid arthritis.

The pre-incubated cells were activated and differentiated towards T_H_1 using Dynabeads™ Human T-Activator CD3/CD28 (1 bead/cell) (Dynal AS, Lillestøm, Norway), 5 µg/ml recombinant human IL-12p70, 10 µg/ml recombinant human IL-2 and 5 mg/ml anti-IL-4 antibodies (clone MAB204; all from, Bio-Techne, Minneapolis, USA). P4 was added together with the activation and differentiation mixture to the cells pre-incubated with P4 cells. The cells were incubated at 37°C, with 5% CO_2_ in RPMI 1640 media containing L-glutamine, 10% FBS and 1% Penicillin/Streptomycin (all from Gibco^®^, ThermoFisher Scientific). Cells were either cultured in 6-well (for RNA-seq) or 96-well plates (for ATAC-seq) in biological triplicates at a density of 1 million cells/ml. Cells were subsequently harvested at 0.5, 1, 2, 6 and 24 hrs and processed for RNA-seq and ATAC-seq. An overview of the study design is shown in **Figure 1**.

### RNA Sequencing

RNA was isolated using the ZR-Duet DNA/RNA kit (Zymo Research, Irvine, USA) and stored at -80°C. The RNA quality was controlled using Agilent RNA 6000 Nano Kit (Agilent Technologies, Santa Clara, CA, USA) on an Agilent 2100 Bioanalyzer instrument (Agilent Technologies). RNA integrity numbers (RIN) were >9 in all samples. RNA library preparation and the subsequent RNA-sequencing were carried out by the Beijing Genomics Institute (https://www.bgi.com/global/). Library preparation was performed using the TruSeq RNA Library Prep Kit v2 (Illumina, San Diego, USA). Each sample was sequenced to the depth of 40-60 million reads per samples with pair end sequencing and a read length of 100bp on an Illumina NextSeq 2500 or 4000 instrument (Illumina). The transcriptomic data derived from human naïve T_H_ cells differentiated under T_H_1 polarizing (n=9 individuals pooled in 3 biological replicates) has previously been published elsewhere (18). The data can be found on ArrayExpress (https://www.ebi.ac.uk/arrayexpress/), accession number E-MATB-7775.

### Nuclei Extraction for ATAC Sequencing

The ATAC-seq sample preparation was carried out in accordance with the OMNI-ATAC-seq protocol (19), modified to incorporate the Nextera tagmentation reagents (20). After culture, the cells were transferred to DNA low-binding tubes (Eppendorf, Hamburg, Germany) for nuclear extraction. The cells were washed with PBS by centrifugation at 500g for 5 minutes (min). The supernatant was discarded by pipette and the cells resuspended in 50 µl ATAC-seq RSB buffer (10 mM Tris-HCl pH 7.4, 10 mM NaCl and 3 mM MgCl_2_) containing 0.1% NP40, 0.1% Tween-20 (all from Sigma-Aldrich) and 0.01% digitonin (Promega, Madison, WI) and incubated for 3 min. ATAC-seq RSB buffer containing 0.1% Tween-20 was added and the nuclei were pelleted by centrifugation at 500g for 10 min. All steps for the nuclei extraction were carried out on ice and all buffers were pre-cooled to 4°C. Nuclei extraction was immediately followed by library preparation.

### ATAC Sequencing Library Preparation and Sequencing

ATAC-seq library preparation was carried out by resuspending the nuclei pellet in 50 µl transposition mix (25 ml 2X TD buffer, 2.5 µl transposase (both from Illumina, San Diego, CA), 16.5 µl PBS, 0.5 µl digitonin, 0.5 µl 10% Tween-20 and 5 ml Milli-Q water). The reaction mix was incubated for 30 min at 37°C in an Eppendorf Thermomixer Comfort (Eppendorf, Hamburg, Germany) at 1000 rpm. The reaction was stopped through the addition of 250 µl DNA binding buffer from the DNA Clean-up and Concentration kit (Zymo-Research, Irvine, CA) and the DNA purified according to the instructions provided by the manufacturer. The ATAC-seq DNA libraries were amplified and indexed with a NEBNext^®^ Multiplex Oligos for Illumina^®^ Index kit (New England Biolabs Inc, Ipswich, MA, USA) by PCR with the following cycling conditions: 72°C 5 min, 98°C sec, followed by 5 cycles of 98°C 10 sec, 63°C 30 sec, 72°C 1 min. Following PCR DNA concentration was assayed by qPCR and additional PCR cycles were added if necessary. After PCR, the samples were once again purified using the DNA Clean-up and Concentration kit (Zymo-Research). Library quality and concentration was assessed by fluorimetry with a Qubit™ dsDNA HS Assay Kit (Thermo Fisher Scientific) and gel electrophoresis using a Fragment Analyzer (ThermoFisher Scientific). Sequencing of the libraries was carried out on an Illumina NovaSeq 6000 instrument by the National Genomics Infrastructure of Sweden (https://www.scilifelab.se/facilities/ngi/).

### RNA Sequencing Data Analysis

Summary of the data analysis can be found in **Figure S1**. Sample quality was assessed with FastQC (Babraham Bioinformatics). Trimming of adapters and clean-up of low-quality reads was performed using TrimGalore! (https://www.bioinformatics.babraham.ac.uk/projects/trim_galore/). Paired-end reads were aligned and mapped to the Ensemble human reference genome GRCh38 (Genome Reference Consortium Human Build 38) using STAR (version 2.6.0c) (21). Transcript assembly and gene quantification was carried out with StringTie (version 1.3.4d) (22) using the GRCh38.90 annotation from Ensemble.

### ATAC Sequencing Data Analysis

Sample quality was assessed with FastQC (Babraham Bioinformatics). To evaluate the ATAC-seq data, the transcription start site (TSS) enrichment was calculated with tssenrich (tssenrich 1.3.0 for Python), which showed an average acceptable level of 5. Sequencing adapters were trimmed using Trim Galore! (https://www.bioinformatics.babraham.ac.uk/projects/trim_galore/). Reads were aligned to Genome Reference Consortium Human Build 38 from RefSeq using Bowtie 2 (23). Peak calling was performed with Genrich (https://github.com/jsh58/Genrich) and reads in peaks were counted with featureCounts from the Subread package (24). Prior to counting of reads in peaks and the calling of footprints, duplicate reads were dropped with Picard MarkDuplicates. Additionally, all alignments were shifted with alignmentSieve –ATACshift from deepTools (25) prior to the counting of reads in peaks.

### Differential Expression Analysis

ATAC-seq peak counts and RNA-seq read counts were both analyzed with maSigPro (version 1.64) (26, 27) in R (version 4.1.1). Prior to the application of maSigPro lowly expressed genes was filtered out by dropping genes who did not exceed 1 read count per million reads mapped (cpm) in at least three samples. Peak and gene counts were normalized in respect to library size through the normalize function from DeSeq2 (28). Peaks were divided into promoter (defined as within 3,000bp from a TSS) and distal peaks (any other peak) prior to differential expression analysis by annotating the peaks to genes with ChIPseeker (29). Promoter and distal peaks were analyzed separately. Peaks and genes were considered as significantly differentially accessible (ATAC-seq) or differentially expressed (RNA-seq) if they had a False Discovery Rate (FDR) < 0.05 from maSigPro and an absolute log_2_fold change > 0.5.

### Peak and Gene Integration by Correlation

The ATAC-seq and RNA-seq data was integrated by correlation to identify changes in the chromatin that was also reflected at the transcriptomic level. Peaks were overlapped to the GRCh38.90 reference annotation from Ensemble with the findOverlaps function from GenomicRanges (30) in R. Pearson correlation was then determined with the base function cor by comparing the log_2_fold change over control (resting naïve, timepoint 0) of each gene and peak in the two data sets. The average correlation and standard deviation were calculated and the gene with the highest (absolute) average correlation was assigned to the peak. Peaks with opposing or highly variable correlation were then dropped by dropping all peaks with a standard deviation above 0.3. Finally peaks and genes not significant by FDR < 0.05 (as defined from maSigPro differential analysis) were also dropped. Significant genes were then determined based on ATAC-seq log_2_fold change (> 0.5) for each time point and the genes assigned to the peaks overlapped with intersect to get the overlapping genes between the T_H_1 response and P4 responses. Gene ontology (GO) pathway enrichments were carried out with compareCluster from the package clusterProfiler (31, 32).

### ATAC Sequencing Footprinting, Motif Finding and Enrichment

Footprints were called using the wellington algorithm from pyDNase (33) with the following parameters, -sh 7,36,1 -fp 6,41,1 -A -fdr 0.01 -fdriter 100 -fdrlimit –30. To achieve the recommended sequencing depth (100*10^6 reads per sample) for footprinting the ATAC-seq read alignments files for each time point were merged using samtools v1.6 prior to the calling of footprints. The footprints were then matched to motifs using findMotifsGenome.pl from HOMER (34) with the parameters -size given and -find. Motif enrichment over the control state (resting naïve T-cells, time point 0) was performed in parallel with findMotifsGenome.pl with the parameters “-size given” and -keepOverlappingBg. For this, motif enrichment footprints from both the T_H_1 and T_H_1+P4 treated time series were used for each time point. Overlapping footprints were merged with bedtools merge (version 2.27.1). The motif analysis of the footprints in the peaks of genes overlapping between upregulated in T_H_1 and downregulated in P4 was analyzed in the same way and against the same background. Tables were merged for downstream analysis in R using the package pandas in python (version 3.6).

### SNP Enrichment Test

Disease single nucleotide polymorphisms (SNPs; P < 10^-5) in linkage disequilibrium (LD; LD threshold = 0.8, acquired using SNiPA https://snipa.helmholtz-muenchen.de/snipa3/) from each disease (**Table S1**) were mapped to the closest gene using ChIPseeker. Based on this mapping, genes were then considered as SNP promoter-associated if they had a disease SNP within 3,000bp from TSS or SNP distal-associated if within 100, 000bp from TSS (same as the definition of promoter and distal peaks). The SNP-associated genes were then overlapped to genes linked to peaks as established previously by correlation and the number of SNP to gene associated peaks in the peaks linked to the union of overlapping genes were compared to the number of SNP gene associated peaks overall. Promoter and distal peaks were analyzed separately. Disease enrichment was performed with Fisher’s exact test where a p-value of <0.05 was considering statistically significant. We validated the TF *JUN* as a regulator of MS by calculating the enrichment of SNPs associated with MS in *JUN* binding regions. The enrichment of MS SNPs was calculated using the function permTest (permutations = 10000) from the R package regioneR (30) which performs a permutation test. SNPs significantly associated with MS, using a nominal p < 10^-5. We used all SNVs in linkage disequilibrium with the MS-associated SNPs computed using SNiPA (r2 > 0.8, 1000 Genomes Phase 3 v5 variant set, European population). There were 11,249 SNPs in linkage disequilibrium with the 710 MS SNPs. *JUN* binding sites from *JUN* ChIP-seq on human were downloaded from the ENCODE portal (https://www.encodeproject.org/). We used conservative idr thresholded peaks with identifiers ENCSR000EFS, ENCSR000FAH. Common SNVs in NCBI dbSNP Build 153 with RegulomeDB rank scores were downloaded from RegulomeDB (https://regulomedb.org/). SNVs and *JUN* binding regions were annotated with promoter regions using the function annotatePeak from the R package ChIPseeker.

### Target to Transcription Factor Network

A network of disease-associated target genes and transcription factors was created based on the MS- and RA-associated peaks (**Table S2**). First, each peak in the union of promoter and distal peaks for the diseases was matched to their corresponding footprints and possible matching motifs at each time point through the findOverlaps function from the GenomicRanges packages (35) in R. The motifs for each footprint were then filtered based on the bit score, as determined by HOMER during the motif matching. Only the motif with the highest score was kept for each footprint. This information was then condensed for each peak to motif (TF) interaction in each disease and a bias score was calculated for each interaction. The bias score was calculated by subtracting the number of times the interaction was detected in the T_H_1 and T_H_1+P4 samples respectively, where a negative score indicates a bias towards T_H_1+P4 whereas a positive score is biased towards T_H_1 alone. Only target genes and TFs with a negative bias score were included in the network. Disease-association for the upstream TFs was done using DisGeNet (36). Visualization of the network was done in the open-source bioinformatics visualization software Cytoscape (version 3.8.2; Cytoscape Team).

## Results

### Early T_H_1 Differentiation Involves Chromatin and Transcriptomic Changes in Central Transcription Factors and Genes

**Figure 2:**
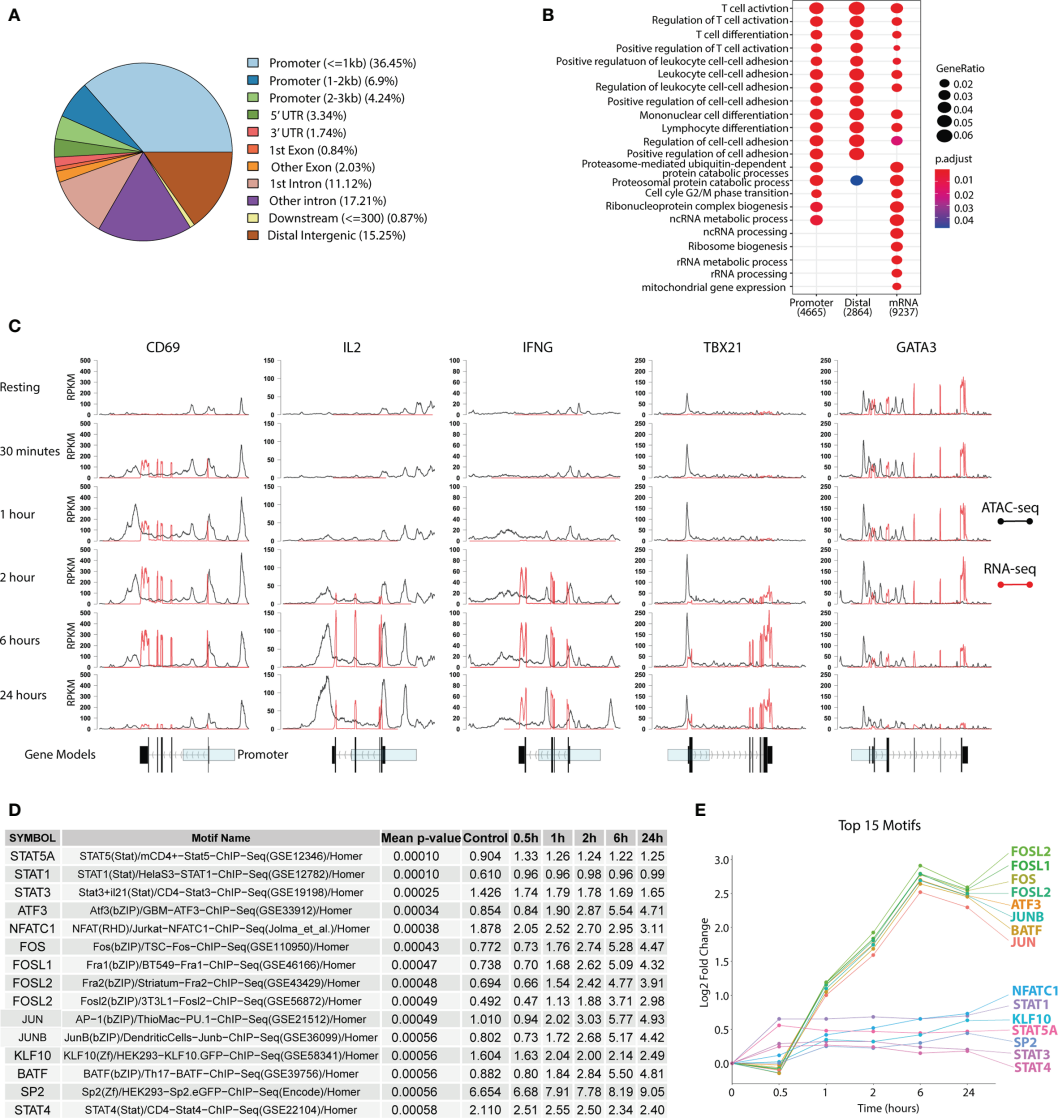
Changes in the chromatin and transcriptomic landscape during T_H_1 differentiation. Primary naïve human CD4^+^ T cells were activated and differentiated towards T_H_1 using CD3/CD28 Dynabeads™, IL-12p70, IL-2 and anti-IL-4 antibodies. ATAC-seq and RNA-seq were used to assess chromatin and transcriptomic changes respectively. **(A)** Peak annotation to genomic elements based on the peaks from the ATAC-seq. **(B)** GO pathway enrichment of differentially accessible or expressed genes over the whole T_H_1 time series. **(C)** Accessibility (black for ATAC-seq) and expression (red for RNA-seq) shown as RPKM for *CD69*, *IL2*, *IFNG*, *TBX21* and *GATA3* over time. Gene models of the genes with exons (black) and the promotor region (transparent blue) are displayed below. **(D)** Table of the top 15 most highly enriched motifs over the T_H_1 time course with the percentage of total discovered motifs stated for each TF. Geometric mean of adjusted p-values over the whole time series are shown in the table. **(E)** Gain in percentage of total discovered motifs (compared to baseline) for the top 15 motifs presented as log_2_fold change over the time series. TF, transcription factor; T_H_1, T helper 1 cells; RPKM, Reads per kilo base per million mapped reads.

We performed ATAC-seq in combination with RNA-seq to investigate the global chromatin landscape and the associated gene expression profiles during the earliest stages of human T_H_1 differentiation (0.5-24 hrs) using isolated naïve CD4^+^ T cells (**Figure 1**). A total of 41,466 peaks were found of which 47.6% resided in promoter regions (≤3,000 bp from TSS), while the rest were assigned to exonic, intronic and intergenic regions (**Figure 2A**), hereby referred to as distal peaks. Using maSigPro (26, 27) to determine significantly differentially expressed peaks and genes over time, we found 23,103 peaks, out of which 7,215 were located in the promoter and 15,888 in distal peaks, and 12,492 genes to be differentially accessible or differentially expressed over the course of the time series (FDR<0.05). By associating the peaks to their closest genes, a clear T-cell stimulatory response could be seen as evident by the enrichment of T-cell activation pathways (**Figure 2B**). Furthermore, a gain in promoter ATAC-seq read coverage and increase in RNA-seq exon coverage could be seen for several prototypic T-cell activation markers, first for the early surface marker *CD69* at 0.5h followed by the cytokines *IL2* and the T_H_1-associated *IFNG* at 2h. Consistent with the known expression timing for CD69, expression and promotor accessibility peaked at 6h and was almost completely lost by 24h. Concurrently gain was also observed for the T_H_1 master regulator *TBX21* at 2h with the gain peaking at 6h. As expected, this peak in gain of ATAC-seq and RNA-seq read coverage of *TBX21* coincided with the loss of the T_H_2 master regulator *GATA3* (**Figure 2C**), indicating that the chromatin and transcriptomic landscape is becoming more poised towards T_H_1.

To further investigate the regulatory landscape during T_H_1 differentiation, transcription factor (TF) footprints were called in all peaks for each time point using the Wellington algorithm (33) and mapped to TF motifs using HOMER (34). The top 15 enriched TF motifs over time were primarily connected to T-cell activation and differentiation, such as *STATs* and *FOS*. Furthermore, this analysis showed an intriguing pattern where the *STATs* were enriched early (0.5 h) and their fold change peaked at 1 h, while activation markers such as *FOS* and *JUN* were first enriched at 1 h and peaked at 6 hrs, before slightly decreasing by 24 hrs (**Figures 2D, E**). This corroborates well with *STAT*s being the earliest T-cell activation TFs (14), followed by other clusters of TF activation waves.

### Progesterone Dampens T Cell Responses Induced During T_H_1 Differentiation

T_H_1 cells are implicated in several autoimmune diseases that are known to be modulated during pregnancy (37) when P4 levels are high, which promoted us to further investigate how P4 affects T_H_1 differentiation. To this end, naïve T_H_ cells cultured under T_H_1 polarizing conditions alone were compared to T_H_ cells polarized in the presence of P4 (**Figure 1**). As we have previously shown that P4 induces large transcriptomic changes during T cell activation (10), we wanted to gain further functional insights into underlying gene regulatory machinery. We therefore used correlation between ATAC-and RNA-seq to identify more robust changes induced by P4 that are transmitted across both omics. To map changes in the chromatin state to changes in gene expression, we correlated the change in peaks over time to the change of proximal gene expression over time. For this purpose, promoters and distal peaks were correlated to genes within 3 kbp and 100 kbp respectively as to represent short and long range interactions as defined by Chen et al. (38) for T_H_1 differentiation in the presence or absence of P4. This resulted in a set of 5,284 promoter correlating to 4,172 genes and 8,771 distal peaks correlating to 3,926 genes. For the distal elements, regression analysis showed that base-pair closeness was not significantly associated with mRNA correlation (data not shown). Moreover, for each distal peak we tested on average 3.14 different genes and in 48.4% we mapped the peak to a gene that was not closest to the peak, while the promoters were tested for 1.24 genes on average resulting in 11.6% of the promoters to be mapped to non-neighboring genes (**Tables S3**, **S4**). P4 has previously been shown to dampen T-cell activation and we also observed the largest increase in percentage overlap over time between genes upregulated during T_H_1 differentiation and downregulated by P4, increasing from 17% at 30 min to 86% overlap after 24 hours in promoter genes and from 15% to 74% in distal genes (**Figure S2**). Therefore, we decided to focus on the dampening effect of P4 on T_H_1 differentiation.

**Figure 3:**
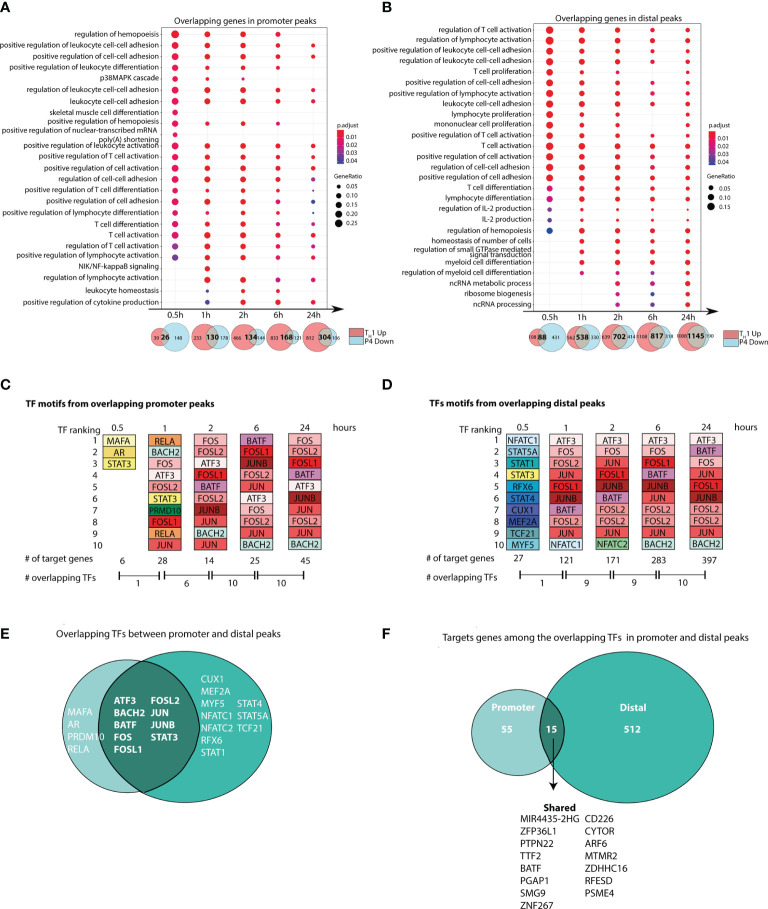
Progesterone downregulates T-cell responses induced during T_H_1 differentiation. Naïve primary human CD4^+^ T cells were differentiated in the presence or absence of P4 to evaluate the influence of P4 on T_H_1 differentiation. Correlation between ATAC-seq and RNA-seq was performed to identify changes that were present in both omics. **(A, B)** Gene Ontology pathway enrichment analysis of the overlapping genes between peaks that were upregulated during T_H_1 differentiation and downregulated by P4. Venn diagrams shown below highlight the overlapping genes between the genes upregulated in T_H_1 and downregulated with P4, where the intersection was used for the gene set enrichment. **(C, D)** Top 10 transcription factors derived from motif enrichment analysis of the intersecting peaks between T_H_1 up and P4 down at each time point. The TFs are colored in the same color among the same TF and between promoter and distal. A TF can appear more than ones for each time point since different motifs from the same TF are present. Number of target genes regulated by these TFs and the number of shared TFs between the time points are given below. All TFs are significantly enriched compared to time point 0 (resting; p < 0.05) at each respective timepoint they appear. **(E, F)** Venn diagram showing the number of overlapping and unique target genes and TFs enriched in the promoter and distal peaks comparing genes upregulated during T_H_1 and downregulated with P4. GO, gene ontology; P4, progesterone; TF, transcription factor; T_H_1, T helper 1 cells.

Pathway enrichment of these overlapping genes showed enrichment of pathways related to T-cell activation and differentiation across the time series in both promoter and distal peaks, further supporting an inhibitory effect of P4 on T-cell activation (**Figures 3A, B**).

Analysis of the upstream regulators of these overlapping genes showed that the top 10 TF motifs belonged mainly to core set of TFs (*ATF3, BATF, BACH2, FOS, FOSL1, FOSL2, JUN, JUNB*). These were consistently among the top 10 TF motifs at most time points in both promoter and distal peaks (**Figures 3C–E** and **Tables S5**, **S6**). In contrast, at 30 min, a completely different set of TFs, including *STAT1*, *STAT3*, *STAT4* and *STAT5A* appeared among the top 10. Over time, the core set of TFs regulated more and more target genes over the time series. At 6 and 24 hrs, these TFs regulated more than 30% of the target genes associated to distal peaks and around 15% of the target genes associated to promoter peaks (**Figures 3C, D**). Overlap of all peak-associated genes and top 10 TF motifs across the time series highlighted that several known TFs of T-cell activation and their targets genes were regulated in both promoter and distal peaks affected by P4 (**Figures 3E, F**).

### P4 Affects Disease-Associated Genes Involved in T_H_1-Mediated Diseases

**Figure 4:**
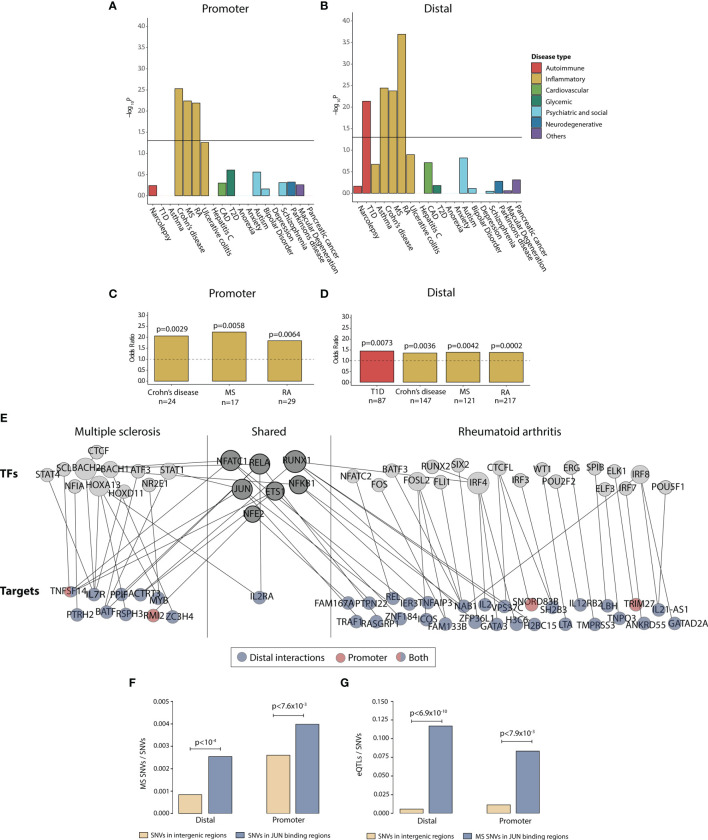
Progesterone downregulates disease-associated genes in immune-mediated diseases. Enrichment of disease SNP-associated genes in the promoter and distal peaks that were overlapping between peaks upregulated during T_H_1 differentiation and downregulated with P4. **(A, B)** -log_10_ p values for disease enrichment in the overlapping promoter and distal peaks. The promoter and distal peaks were combined over the whole time series. Disease enrichment was performed with Fisher’s exact test where a p-value of <0.05 was considering statistically significant. **(C, D)** Odds ratio for enrichment of SNP-associated peaks in Crohn’s disease, MS, RA and Type 1 diabetes with the number of overlapping genes (annotated from the SNPs) are denoted below. **(E)** Network of the genes and TFs present in the overlapping promoter and distal peaks for MS and RA. Shared TFs and genes are present in both diseases. The target genes have been colored based on if the gene is associated to the promoter peaks (red), distal peaks (blue) or both (blue and red). Some of the genes were denoted both as TFs and targets. **(F)** Proportion of MS-associated SNPs among common SNVs in intergenic regions compared to JUN binding sites, both for the distal and promoter regions. Statistical differences were determined using permutation test (10,000 permutations). **(G)** Proportion of eQTLs among SNVs in intergenic regions compared to proportion of eQTLs among MS-associated SNPs in JUN binding sites, both for the distal and promoter regions. Fisher’s exact test was used to determine statistical differences. CAD, coronary artery disease; MS, multiple sclerosis; RA, rheumatoid arthritis; SNP, single nucleotide polymorphism; SNV, single nucleotide variant.; eQTL, expression quantitative trait locus; T1D, Type 1 diabetes; T2D, Type 2 diabetes; TF, transcription factor; T_H_1, T helper 1 cells.

To get a more complete understanding of how P4 can be involved in modulating disease-associated changes, we investigated how the changes induced by P4 during T_H_1 differentiation were related to known disease-associated SNPs. For this purpose, we used the union of all peaks correlated to the genes that were upregulated during T_H_1 differentiation and downregulated with P4 over the entire time series, resulting in a total of 599 promoter and 3490 distal peaks. Using established disease-associated SNPs from different types of diseases ranging from psychiatric and social to autoimmune (**Table S1**), we found that the peaks were significantly enriched for peaks correlated to SNP-associated genes for autoimmune and immune-mediated disease (**Figures 4A–D** and **Table S7**), whereas no other diseases were found to be enriched.

### P4 Downregulate the Activity of Central T-Cell Activation TFs, Particularly *JUN*

Classical T_H_1-associated diseases like MS and RA have been shown to markedly improve during pregnancy, particularly during the third trimester, whereas a temporary rebound effect after pregnancy has been observed, coinciding with high and low P4 levels, respectively. We therefore focused specifically on disease-associated changes related to these two diseases. To increase the functional insight of the MS- and RA-associated changes in relation to P4, we constructed a network of the affected disease genes and their upstream disease-associated TFs (**Table S2**). As expected, most peaks correlating to disease SNP-associated genes were found among the distal peaks (**Figure 4E**). Interestingly, among the target genes, the T-cell activation marker *IL2RA* was common between the two diseases. Several TFs were also shared between the two diseases, including *JUN, NFKB1, RUNX1* and *RELA*, that regulated both MS- and RA-associated disease genes (**Figure 4E**), whereof *JUN* was in our previously derived core TFs that we predicted to significantly regulate P4 induced gene expression through promoters and distal elements. To justify the potential upstream relevance of *JUN* in MS we identified two previously published ChiP-seq studies in immune-related cell lines (39, 40), which were combined to get validated peaks of *JUN*, both in promoters and distal elements respectively. Again, we found MS-associated SNPs to be highly enriched among the promoter (permutation test P<7.6 x 10^-3) and distal (P<10^-4) bindings elements of *JUN* (**Figure 4F**). Lastly, using RegulomeDB annotation we found those SNPs to be highly enriched as expression quantitative trait loci (promoter P<7.9 x 10^-3; distal P<6.9 x 10^-3; **Figure 4G**). In summary, this supports the hypothesis of a key aspect of P4 autoimmune relevance is to downregulate the activity of central T-cell activation TFs, particularly *JUN*.

## Discussion

We performed a detailed investigation on the effect of P4 on T_H_1 differentiation in relation to T_H_1-mediated diseases, whose disease activity is altered during pregnancy when P4 levels are high. Interestingly, P4 altered chromatin accessibility in both promoter and distal peaks associated to genes involved in T-cell activation, opposing the changes induced during T_H_1 differentiation. Furthermore, motif analysis identified several TFs, such as *ATF3*, *FOS*, *JUN* and *JUNB*, as central upstream regulators of these changes. The changes induced by P4 were significantly enriched for peaks associated to genes for several immune-mediated diseases, including MS and RA. Further analysis of disease-related changes associated to MS and RA revealed several shared upstream TFs between the diseases. *JUN*, a central TF during T-cell activation, appeared as one of the shared disease-associated TFs that also had motifs in both promoter and distal peaks affected by P4. Our findings provide new knowledge of how P4 affects the gene regulatory landscape during T_H_1 differentiation and offer valuable insights into central changes that could be responsible for the pregnancy-induced modulation of T_H_1-mediated diseases.

The use of ATAC-seq enables interrogation of non-coding regions of the DNA, such as in promoters and other distal regulatory elements, which is not captured by transcriptomics alone. Nearly 90% of GWAS-associated SNPs lie within non-coding regions (41) and hence, to be able to capture the functional implications of these SNPs, more studies investigating the gene regulatory landscape in diseases are needed. This was recently highlighted by Liu et al. where accessible chromatin regions were enriched for diseases-associated SNPs and could predict degree of diseases in patients with skin fibrosis (42). However, analysis of this data is complex as the non-coding regions might act on a distance directly regulating distal genes, which also may vary across cell types making existing chromatin maps less relevant in differentiation studies. In this study, we mapped long range chromatin changes by highest absolute correlations using paired transcriptomic profiles of all genes within 3,000 and 100,000 base pairs for promoters and distal regions respectively. Not surprisingly in about 50% of the distal regions this resulted in a mapping to a non-neighbouring gene as enhancer elements often act at a long distance, and we found no association between distance to TSS and absolute correlation within 100,000 base pairs. To justify our mappings and increase the validity of our findings, we throughout the paper used corroboration of the promoter and distal genes.

P4 is the one of the major pregnancy hormones and has historically been proposed as a candidate involved in the shift from determinantal T_H_1 responses to more T_H_2-skewed immune response that has been suggested to underlie successful pregnancy (43). The most prominent changes induced by P4 was counteracting changes induced during T_H_1 differentiation, where genes upregulated during T_H_1 differentiation were downregulated by P4, supporting a modulatory role of P4 during T_H_1 differentiation. Indeed, these changes were highly enriched in pathways involved in T-cell activation, the first crucial step involved in T-cell lineage commitment, both in peaks associated to promoter and distal elements. The dampening effect of P4 on T-cell activation are in line with previous findings (10, 11, 44), although here we provide a more fine-grained time series analysis of how P4 affects T-cell activation, in particularly related to T_H_1 differentiation. More in-depth analysis revealed that the major TFs regulating these changes were dominated by well-known TFs involved in T-cell activation such as *BATF*, *FOS, FOSL1, FOSL2, JUN* and *JUNB.* This was evident not only from the promoter peaks but also in the distal regulatory elements. These are all members of the AP-1 transcription factor family, a key component of T-cell activation (45). Indeed, AP-1 activity has been shown to be the major TF involved in the remodeling of the chromatin during T-cell activation and sites in AP-1 are highly overlapping with risk loci for immunological diseases, particularly in MS (46). It is therefore striking that P4 seemingly affects the binding motifs of several TFs of AP-1 and points towards a possible molecular mechanism by how P4 can dampen T-cell activation, which we have not observed previously (10).

The modulation of several immune-mediated disease during pregnancy, which correlates with P4 levels, makes P4 a very attractive candidate that could at least partly be responsible for the beneficial effects of pregnancy on disease. In fact, P4 treatment before onset and during ongoing disease reduces clinical severity and shows neuroprotective effects in the MS animal model experimental autoimmune encephalomyelitis (EAE) (47–50). We have previously shown that P4 affects disease-associated genes related to several immune-mediated diseases (10), including MS and RA. In agreement with these findings, we found that the downregulatory changes induced by P4 during T_H_1 differentiation were also significantly enriched for diseases such as MS and RA. More in-depth analysis showed that several upstream TFs were shared between MS and RA such as *JUN*, *ETS1*, *NFATC1* and *NFKB1*. *NCATC1* controls proliferation and survival of peripheral lymphocytes and *NFATC1* deficiency has been shown to ameliorate the course of EAE and has been suggested as a potential treatment option (51). *ETS1* is a functional co-factor of Tbet and important for the differentiation and function of T_H_1 cells (52). The TF *JUN* was not only shared between the two diseases but was also present among the TF motifs associated to both promoter and distal peaks and was shown to be significantly enriched in MS-associated SNPs, supporting its importance in disease. Interestingly, one downstream target gene was shared between the diseases, *IL2RA.* IL-2RA has an undisputable role during T-cell activation as a part of the receptor for IL-2, an important initial growth factor during early T-cell activation (53). Still, it should be noted that IL-2RA is also important for regulatory T cells, which has placed IL-2RA at the border between immunity and tolerance (53). However, an initial dampening of IL-2RA by P4 during the earlier phases of T_H_1 differentiation would most likely impede T-cell activation as it would render the cells less responsive to IL-2. It is evident that many of the downregulatory disease-associated effects related to P4 are on major players involved in T-cell activation. Indeed, during pregnancy, alloreactive T cells would need to be kept under control to limit potential detrimental immune responses towards the semi-allogenic fetus and aberrant T-cell activation is a prominent feature of both MS and RA.

One major limitation of this study is that we only assessed the effect of P4 during the earliest phases of T_H_1 differentiation where activation was primarily affected by P4, which limits our ability to draw major conclusions about the effect of P4 on the T_H_1 differentiation itself. The enrichment of genes associated to MS, RA, T1D and Crohn’s disease suggests a more T_H_1-specific effect although it should be noted that the pathogenesis for several of these diseases is not solely attributed to T_H_1. In MS, classically considered a T_H_1-mediated disease, T_H_17 also plays an important role (54, 55) although recent studies suggest that T_H_1/T_H_17 cells are the major pathogenic cell type (56, 57). At these early stages of the polarization, disentangling the effect of P4 on T_H_1 and T_H_17 is difficult as activation predominates. Still, the early effect on STATs involved in T_H_1 differentiation and the enrichment of genes associated only to more T_H_1-associated diseases argues for a potential T_H_1-specific effect of P4 even during these earliest events although the long-term impact of this will need to be further investigated. Cytokine production has been shown to be affected even after several days of culture with P4 (6, 58, 59), which suggests that the effects of P4 on T cells are long-lasting and persisting. Capturing the early effects of P4 are central since the T-cell activation sets the stage for the ensuing T-cell differentiation. Moreover, the early effects might be the most critical as they can lead to a cascade of downstream effects. This principle was also previously used by us to determine upstream disease-relevant TFs, although we then started from 6h instead of 30 mins (60). Herein, this led to the consistent prediction of *JUN* as a P4 regulated TF and key upstream regulator of MS associated SNPs (61), which our independent ChiP-seq analysis further confirmed.

In summary, we show that P4 alters the gene regulatory landscape during early T_H_1 differentiation by modulating the activity of several central TFs and target genes involved in T-cell activation. The changes induced by P4 were enriched for disease-associated changes related to several immune-mediated diseases, including MS and RA, diseases that significantly improves during pregnancy, coinciding with high P4 levels. Our results further support the importance of P4 in the immunomodulation during pregnancy and in disease. We believe our findings speaks strongly in favor of investigating the potential benefit of using P4 or P4-derivates as a potential treatment option in T-cell mediated diseases.

## Data Availability Statement

The datasets presented in this study can be found in online repositories. The names of the repository/repositories and accession number(s) can be found below:

https://www.ebi.ac.uk/arrayexpress/, E-MTAB-7775

https://www.ebi.ac.uk/arrayexpress/, E-MTAB-10423

https://www.ebi.ac.uk/arrayexpress/, E-MTAB-10444

https://gitlab.com/Olof_Rundquist/th1_p4_atac_and_rna_seq.

## Ethics Statement

The use of blood from blood donors does not require a specific ethical approval in accordance with the local legislation and institutional requirements. The participants provided their written informed consent for research use (besides medical use) of the donated blood in accordance with the Declaration of Helsinki.

## Author Contributions

OR collected samples and performed all experimental work. OR and SH performed the bioinformatics analysis. CN, SH, MJ, and MG contributed to the study design and overall supervision of the study. OR, SH, and MG prepared the figures and writing of the manuscript. All authors have read and approved the final manuscript.

## Funding

This study was funded by the Swedish Foundation for Strategic Research (SB16-0011).

## Conflict of Interest

The authors declare that the research was conducted in the absence of any commercial or financial relationships that could be construed as a potential conflict of interest.

## Publisher’s Note

All claims expressed in this article are solely those of the authors and do not necessarily represent those of their affiliated organizations, or those of the publisher, the editors and the reviewers. Any product that may be evaluated in this article, or claim that may be made by its manufacturer, is not guaranteed or endorsed by the publisher.
