# Supplementary material for: Progesterone Inhibits the Establishment of Activation-Associated Chromatin During TH1 Differentiation
Source: Front Immunol. 2022 Feb 2;13:835625. doi: 10.3389/fimmu.2022.835625 (PMC8848251; doi:10.3389/fimmu.2022.835625)
Supplement: Supplementary file 1 [file DataSheet_1.pdf]

# *Supplementary Material*

## 1 Supplementary Figures and Tables

### 1.1 Supplementary Figures

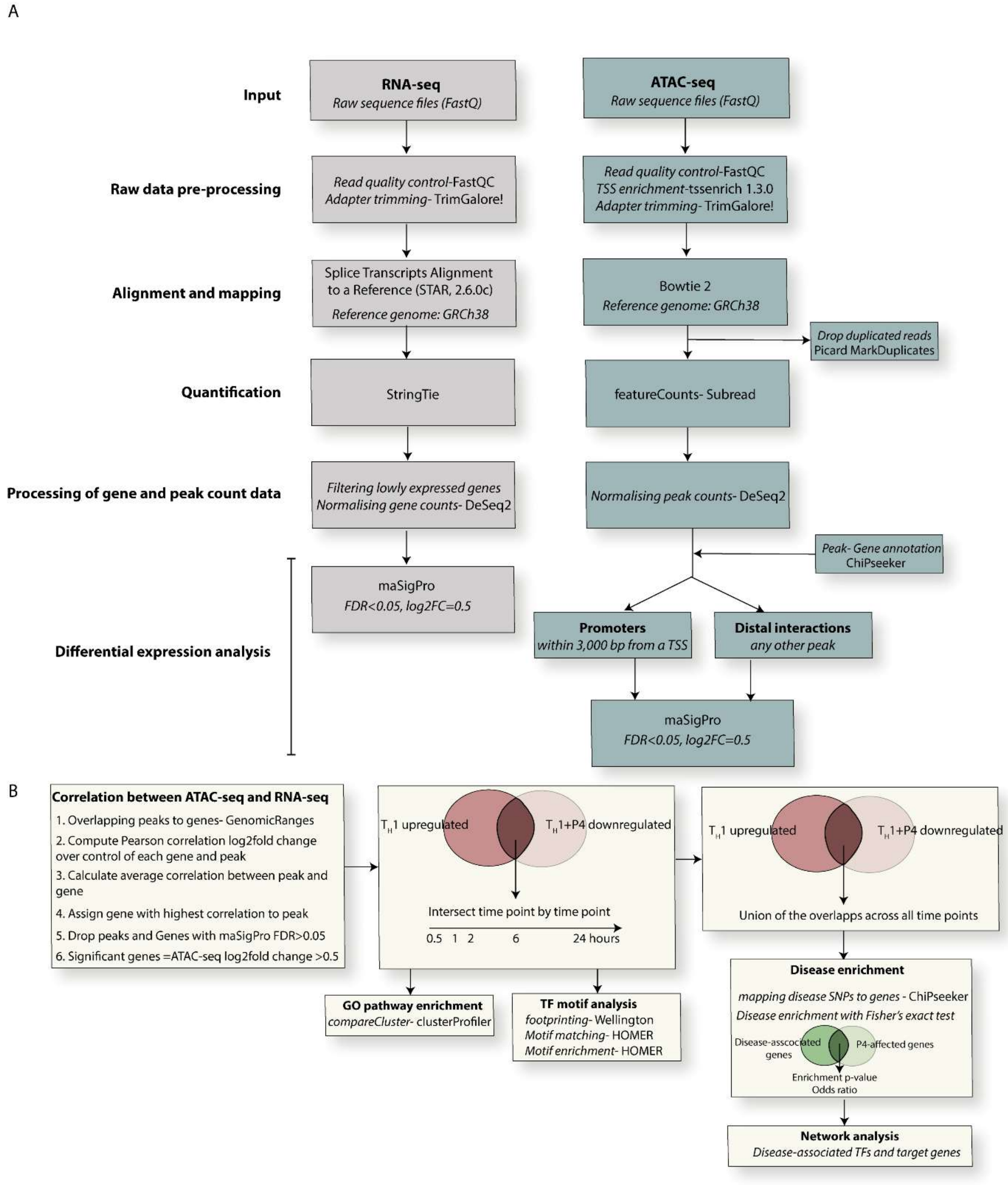

**Figure S1. Data analysis pipeline.** Pipeline for the workflow for ATAC-seq and RNA-seq analysis. Each steps gives a brief description and if needed, the specified R package used (A) Raw data analysis to differentially accessible peaks and genes. (B) Downstream analysis of peaks and genes derived from correlating the ATAC- and RNA-seq data. Further details regarding the methods can be found in the materials and methods. P4= progesterone, SNP = single nucleotide polymorphism, $T_H1$= T helper 1 cells.

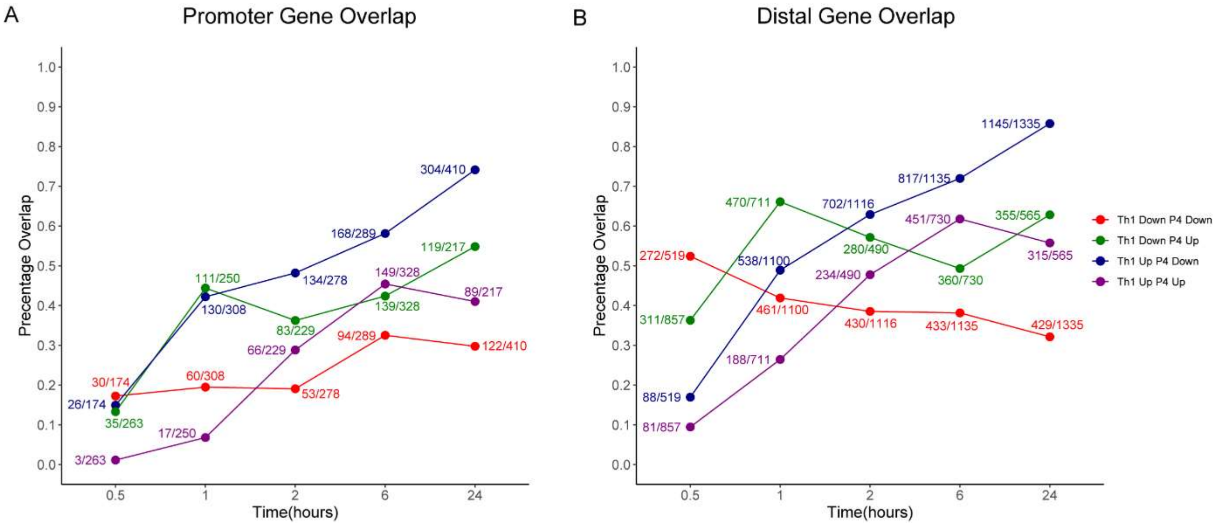


**Figure S2. Gene overlap in $T_H1$ *versus* $T_H1$+P4.** The overlap of significantly differentially expressed genes for each time point in $T_H1$ versus $T_H1$+P4. Overlapping genes assigned from (A) promoter peaks and (B) distal peaks shown as line plots. The ratio of P4 affected genes overlapping with respective to the $T_H1$ affected genes are shown at each time point. Pearson correlation was used to determine correlation between the $log_2$fold change over control (timepoint 0) of each peak and gene in the ATAC-seq and RNA-seq data respectively. The gene with the highest (absolute) average correlation was assigned to the peak. Significant genes were then determined based on ATAC-seq $log_2$fold change (>0.5) for each time point. P4= progesterone, $T_H1$= T helper 1 cells.

## 1.2 Supplementary Tables

**Supplemental Table S1. Overview of the different GWAS data sets used in Figure 4 and SNPs used from each disease.** Disease SNPs (P < 10^-5) in linkage disequilibrium (LD) (LD threshold = 0.8, acquired from SNiPA) were used for disease-enrichment analysis. Note: Not all data sets are available for public download.

**Supplemental Table S2. Network of RA-and MS-associated targets and transcription factors.** The "Associatin type" displays in which type of peaks, promoter or distal, that the target genes occur. MS= multiple sclerosis, RA =rheumatoid arthritis

**Supplemental Table S3. Peak-to gene correlation for promoter regions.** For each peak the gene with the highest absolute mean correlation was chosen. distanceToTSS describes the distance from the edge of the peak to the transcription start site (TSS) of the gene. If the TSS was within the peak distance was considered as 0. Negative values are upstream, positive values are downstream of TSS. Th1 and P4 columns describe whether the peak (ATAC) or gene (RNA) was significantly changed (maSigPro, FDR < 0.05) during the time series.

**Supplemental Table S4. Peak-to gene correlation for distal regions.** For each peak the gene with the highest absolute mean correlation was chosen. distanceToTSS describes the distance from the edge of the peak to the transcription start site (TSS) of the gene. If the TSS was within the peak distance was considered as 0. Negative values are upstream, positive values are downstream of TSS. Th1 and P4 columns describe whether the peak (ATAC) or gene (RNA) was significantly changed (maSigPro, FDR < 0.05) during the time series.

**Supplemental Table S5. Top 10 transcription factors in promoter peaks over the time series** in between genes associated to peaks that are overlapping between genes upregulated during TH1 and downregulated by progesterone. Log p values for each TF motis is given as well as the rankning of the TF, number of targets and the names of the targets. Related to Figure 3 in the manuscript. Log p values below -3 was considered as statistically significant. ns= non significant

**Supplemental Table S6. Top 10 transcription factors in distal peaks over the time series** in between genes associated to peaks that are overlapping between genes upregulated during TH1 and downregulated by progesterone. Log p values for each TF motis is given as well as the rankning of the TF, number of targets and the names of the targets. Related to Figure 3 in the manuscript. Log p values below -3 was considered as statistically significant. ns= non significant

**Supplemental Table S7. Odds ratio and p-values for the disease enrichment** in overlapp between the peaks upregulated during TH1 and downregulated with P4. The observed overlapping SNPs were mapped to the nearest gene using ChiPseeker. Significant p-values are highlighted in bold. The total amount of peaks observed in the intersect between up Th1 and down with P4 was used for the enrichment analysis which consisted of 5284 promoter peaks and 8771 distal peaks. This table relates to Figure 4.
